# Creating Complexity

**DOI:** 10.3762/bjoc.9.220

**Published:** 2013-09-16

**Authors:** Donald Craig

**Affiliations:** 1 Department of Chemistry, Imperial College London, South Kensington Campus, London SW7 2AZ, UK

The word synthesis is derived from the ancient Greek word, σύνθεσις, or ‘placing with’, and means literally the combination of two or more entities that together form something new [1]. Therefore, in the realm of chemistry, by definition synthesis entails combining reactants to make a product, and this almost invariably creates complexity.

Organic synthesis is widely regarded as having begun nearly two centuries ago, with Friedrich Wöhler’s 1828 discovery that ammonium cyanate could be converted into urea [2]. Since that seminal finding, chemical synthesis has advanced to extents which must have been unimaginable to its early practitioners. The numerous paradigm-shifts which have taken place throughout the history of synthesis have been driven largely by a single impulse: the fascination of building sophistication from simplicity – in other words, creating complexity.

Chemical synthesis inspires chemists from many different backgrounds, and these scientists are unified in their desire to create and develop methods which enable the creation of complex target molecules from readily available starting materials. In many cases the targeted structure is a naturally occurring, biologically active entity, and over decades of research endeavour, natural products have consistently provided a rich source of inspiration. Away from the natural products arena, organic synthesis chemists are engaged in the creation of novel polymeric materials with a wide range of applications, the conception and fabrication of new supramolecular architectures, and in the exploration of new regions of chemical space in the contexts of drug discovery and development.

As synthesis nears its 200^th^ birthday, and synthesis scientists hone the skill-sets needed to assemble structures of ever-increasing complexity, the definition of complexity is evolving. Traditionally, a complex structure was assembled one step at a time, but as synthesis matures as a discipline, synthesis chemists are searching for quicker, more efficient, more selective, less energy-intensive and more sustainable ways of creating complexity. The search for such improved methods creates additional complexity, whether they involve innovative catalyst design and synthesis, the streamlining of synthesis routes through the omission of protecting groups or the harnessing of intrinsic reactivity, or the shortening of synthesis sequences by carrying out cascade and multicomponent reactions and chemistry in flow.

This Thematic Series brings together a select group of contributors, recognised for their contributions in the areas of catalysis, radical chemistry, stereoselective synthesis and molecular diversity. I thank them warmly for their high-quality contributions, which demonstrate the central role of organic synthesis in all its guises, in the creation of complexity.

Donald Craig

London, July 2013
